# Healthcare utilization after a first hospitalization for COPD: a new approach of State Sequence Analysis based on the '6W' multidimensional model of care trajectories

**DOI:** 10.1186/s12913-020-5030-0

**Published:** 2020-03-06

**Authors:** Alain Vanasse, Josiane Courteau, Mireille Courteau, Mike Benigeri, Yohann M. Chiu, Isabelle Dufour, Simon Couillard, Pierre Larivée, Catherine Hudon

**Affiliations:** 1 Groupe de recherche PRIMUS, Centre de recherche du Centre hospitalier universitaire de Sherbrooke (CRCHUS), 3001 12e avenue nord, Sherbrooke, QC J1H 5N4 Canada; 2 Département de médecine de famille et de médecine d’urgence, Université de Sherbrooke, 3001 12e avenue nord, Sherbrooke, QC J1H 5N4 Canada; 3 École de santé publique de l’Université de Montréal, 7101 avenue du Parc, Montréal, QC H3N 1X9 Canada; 4 Service de pneumologie, Département de Médecine, Université de Sherbrooke, 3001 12e avenue nord, Sherbrooke, QC J1H 5N4 Canada

**Keywords:** (3–10) State sequence analysis, Care Trajectories, Healthcare utilization, Typology, COPD, Optimal matching, TraMineR, Observational study, Data visualization

## Abstract

**Background:**

Published methods to describe and visualize Care Trajectories (CTs) as patterns of healthcare use are very sparse, often incomplete, and not intuitive for non-experts.

Our objectives are to propose a typology of CTs one year after a first hospitalization for Chronic Obstructive Pulmonary Disease (COPD), and describe CT types and compare patients’ characteristics for each CT type.

**Methods:**

This is an observational cohort study extracted from Quebec’s medico-administrative data of patients aged 40 to 84 years hospitalized for COPD in 2013 (index date). The cohort included patients hospitalized for the first time over a 3-year period before the index date and who survived over the follow-up period. The CTs consisted of sequences of healthcare use (e.g. ED-hospital-home-GP-respiratory therapists, etc.) over a one-year period. The main variable was a CT typology, which was generated by a ‘tailored’ multidimensional State Sequence Analysis, based on the “6W” model of Care Trajectories. Three dimensions were considered: the care setting (“where”), the reason for consultation (“why”), and the speciality of care providers (“which”). Patients were grouped into specific CT types, which were compared in terms of care use attributes and patients’ characteristics using the usual descriptive statistics.

**Results:**

The 2581 patients were grouped into five distinct and homogeneous CT types: Type 1 (*n* = 1351, 52.3%) and Type 2 (*n* = 748, 29.0%) with low healthcare and moderate healthcare use respectively; Type 3 (*n* = 216, 8.4%) with high healthcare use, mainly for respiratory reasons, with the highest number of urgent in-hospital days, seen by pulmonologists and respiratory therapists at primary care settings; Type 4 (*n* = 100, 3.9%) with high healthcare use, mainly cardiovascular, high ED visits, and mostly seen by nurses in community-based primary care; Type 5 (*n* = 166, 6.4%) with high healthcare use, high ED visits and non-urgent hospitalisations, and with consultations at outpatient clinics and primary care settings, mainly for other reasons than respiratory or cardiovascular. Patients in the 3 highest utilization CT types were older, and had more comorbidities and more severe condition at index hospitalization.

**Conclusions:**

The proposed method allows for a better representation of the sequences of healthcare use in the real world, supporting data-driven decision making.

## Background

Affecting approximately 250 million people worldwide [1], Chronic Obstructive Pulmonary Disease (COPD) is a common, preventable and treatable disease characterized by progressive airway obstruction, deterioration in lung function and increased mortality. As a leading cause of hospital admissions, COPD is one of the most significant public health concerns [1–3]. With the progression of the disease, acute exacerbations of COPD increase, resulting in an intensive healthcare utilization with frequent physician visits, recurrent emergency department (ED) visits and hospitalizations, and a deterioration of the patient’s health condition and quality of life [3–5]. The course of the illness may also be significantly affected by concomitant chronic conditions such as cardiovascular diseases (CVD), musculoskeletal disorders, diabetes and psychological disorders [3–6]. In this context, healthcare systems are under increasing pressure to propose changes in the care process that could reduce healthcare utilization and improve patient outcomes [4, 7].

Real-world studies are essential to provide empirical evidence for data-driven decision-making in public health [8]. Studies on predictive factors and determinants, as well as healthcare use after a first hospitalization, provide relevant knowledge to improve the early management of COPD and delay further COPD-related adverse events [9–13]. Along with individual and clinical characteristics, the Care Trajectory (CT), defined as the pattern of care use over time, may have an important impact on patient morbidity, mortality and quality of life [7, 14]. Following this assumption, analyzing CTs through real-world observational studies could provide valuable information for evidence-based decision-making.

The increasing volume and availability of medical and administrative data provide opportunities to analyze longitudinal patterns of care use for a specific disease. However, employing appropriate methods to describe and propose a comprehensive visualization of longitudinal patterns of events, without altering the integrity of real patients’ journey through the healthcare system, remains challenging. In recent years, several data mining and statistical approaches have been proposed to extract patterns from sequential data of CTs, such as formal concept analysis [15], latent class analysis [16, 17], neural network [18], multi-state Markov model [12] and exponential proportional hazards mixture model [19]. However, getting the picture of complex temporal event sequences is not straightforward for non-experts, despite the variety of strategies available to develop visual analytic tools and graph-based approaches [20–22]. This emphasizes the need for appropriate methods to describe and visualize sequential patterns of real-world CTs for evidence-based decision-making.

A powerful method for the analysis of longitudinal sequential data has recently risen in healthcare research, although to our knowledge only a few studies have used this approach to describe CTs [23–26]. The State Sequence Analysis (SSA) is largely used in social sciences to describe and visualize longitudinal patterns such as life course or employment status trajectories, where each individual’s trajectory consists of a succession of states and transitions [14, 24, 25, 27–32].

Using the perspective of SSA, a CT consists of a sequence of successive categorical states and transitions, each corresponding to a patient’s record of healthcare use at a given time. As long as a limited number of states are considered, SSA is a powerful method to analyze and visualize CTs. However, given the high number of healthcare use events, which are not mutually exclusive for a large part, the complexity of sequences may lead to noisy plots, or overplotting, a common issue in data visualization [28, 30, 31, 33].

A strategy to address this problem would be applying the “6W” multidimensional model of CTs, which conceptualizes patterns of care use into a comprehensive scheme of six distinct and interrelated dimensions [7]: Patients, with their individual and clinical attributes (“who”), responding to their illness condition and care needs (“why”), will seek healthcare services over different categories of professional care providers (“which”), at ambulatory or inpatient care units and settings (“where”), where they will receive tests and treatments (“what”), at specific periods of time (“when”). According to this model, SSA could be partitioned into reasons for consultation, healthcare professionals, care units and treatments within a specific time frame.

The main objective of this paper is to explore the different patterns of interactions between patients and the healthcare system in the real world: the Care Trajectories. This objective is threefold: 1) to propose a typology of patients’ CTs in the year after their first hospitalization for COPD; 2) to describe and visualize the typology of CTs; and 3) to compare patients’ characteristics according to their type of CT.

## Methods

To achieve this purpose, we propose a “tailored” SSA applying the “6W” multidimensional model of CTs [7]. The analyses focus on the successive interactions in time (“when”) between patients and healthcare services, more specifically: the healthcare units and settings (“where”), the reason for consultation (“why”), and the professional care providers involved (“which”). Several graph-based visualizations of CT types are then proposed with their specific patients’ individual, clinical and environmental characteristics (“who”).

### Design and data sources

This is a population-based retrospective cohort descriptive study. Patients’ data were acquired from the provincial health insurance board (*Régie de l’assurance maladie du Québec*: RAMQ), which provides universal health insurance to Quebec residents, including coverage for physician and hospital services. The RAMQ owns and manages administrative health registers including hospital discharge (MED-ECHO), patients’ demographic information, medical services (including hospital inpatients and outpatients, emergency and primary care clinics), and services provided by physicians and other healthcare professionals at local community service centres (CLSC). The MED-ECHO register contains information on dates of hospitalizations, length of stay, and main and secondary diagnoses (ICD-10). The All Patients Refined Diagnosis Related Groups (APR-DRG) database of the Ministère de la Santé et des Services sociaux (MSSS) includes a variable called NIRRU (relative intensity level of resources used) which measures the level of resources used during a hospitalization, and also a clinical severity index, which indicates the presence of clinical interactive factors, such as comorbidities or complications (degree of physiological decompensation) that influence the intensity of services required for the care provided to the user. The RAMQ demographic database provides information on patients’ age, sex, and date of death. The medical services register provides the date of service, the location, the medical act, and the diagnosis (ICD-9) specific to the medical visit. Using a unique encrypted identifier, patient data from these registers were linked to provide information on demographic characteristics and medical information.

### Studied population

**Fig. 1:**
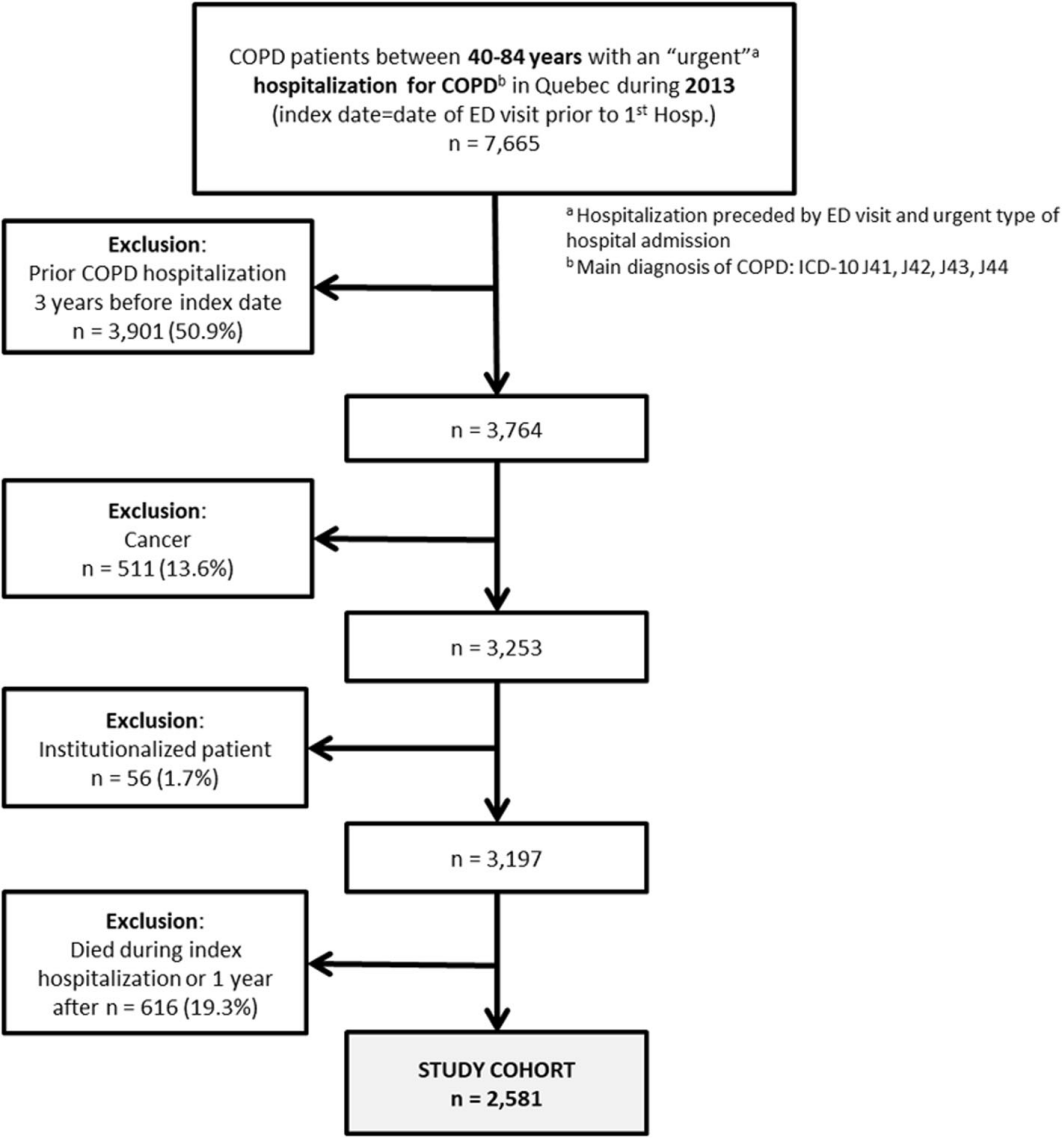
Study cohort flow diagram

The studied population included all patients living in the province of Quebec, Canada, aged 40–84 years, with a first and urgent hospitalization for COPD (main diagnosis ICD-10: J40-J44, J47) between January 1st and December 31st, 2013 (Fig. 1). To increase the likelihood of correct diagnosis, we included only patients aged at least 40 years old and diagnosed with COPD in the 2-year period before the index date (defined as at least two physician ambulatory visits or at least one hospitalization with a secondary diagnosis; ICD-9: 490–494, 496, ICD-10: J40-J44, J47). A hospitalization was considered urgent if the patient was admitted following an ED visit and if the type of hospital admission was considered urgent. The index date refers to the date of the ED visit leading to such hospitalization. To select relatively stable patients with infrequent exacerbations, patients who were hospitalized with a primary diagnosis of COPD in the 3 years before the index date were excluded. We also excluded patient with a diagnosed cancer (as reported in the secondary diagnoses at index hospitalization), and institutionalized patients, identified through the location of the medical services in the 2 years preceding index date. Finally, to describe healthcare use during the one-year follow-up, we excluded patients that died during this period.

### Main variable: CT typology

CTs were defined as sequences in time (dimension “when”) of healthcare utilization associated with the dimensions of the “6W” model [7] and measured in the year after the index date (date of ED arrival to index hospitalization). The main variable consisted of a classification (typology) of CTs (see Statistical analysis section). Information used to define healthcare sequences were: the category of consultation’s care setting (hospital, ED, outpatient clinic, primary care clinic, CLSC) (dimension “where”); the reason of consultation (respiratory disease, CVD, other) (dimension “why”); and the encountered care provider’s category (pulmonologist, cardiologist, internist, other MD specialist, general practitioner (GP), respiratory therapist in CLSC, nurse in CLSC) (dimension “which”).

### Other variables

Demographics and clinical characteristics of the patient (dimension “who”) included: sex; age; physical and mental health conditions, as summarized by a comorbidity index, the severity of index hospitalization; GP affiliation (yes/no); and public prescription drug insurance plan (PPDIP) status. We identified physical and mental conditions using the diagnoses reported in MED-ECHO and in the medical services register in the 2-year period before index date (one diagnosis during a hospitalization or at least two in the medical services register). The comorbidity index selected is proposed by Simard et al., [34] which uses a combination of 31 conditions from the 17 Charlson’s and the 30 Elixhauser medical conditions [35, 36]. The severity of the index hospitalization was measured using variables length of stay, intensity of resources index (NIRRU), and clinical severity index (Weak, Moderate, High, and Extreme). To determine if a patient was affiliated to a GP, we considered all ambulatory visits to GPs (excluding EDs) during the 2-year period before index date. A patient was considered affiliated to a GP if at least 75% of these visits were made to the same GP [37]. If a patient had only one visit to a GP during that period, they were considered affiliated to a GP. The PPDIP status includes four categories: not admissible to PPDIP (individuals with a private drug insurance plan), admissible to PPDIP and age ≥ 65 years with guaranteed income supplement (GIS), admissible to PPDIP and being a recipient of last-resort financial assistance (LRFA), or regular recipient of PPDIP.

Patients’ residential characteristics were also considered as covariables and included the rural-urban characteristic of the residential neighbourhood (dissemination area) (metropolitan: ≥100,000 inhabitants, small town: 10,000–100,000 inhabitants, rural: < 10,000 inhabitants with high to low metropolitan influence) as well as its material and social deprivation quartiles [38].

### Statistical analysis

**Fig. 2:**
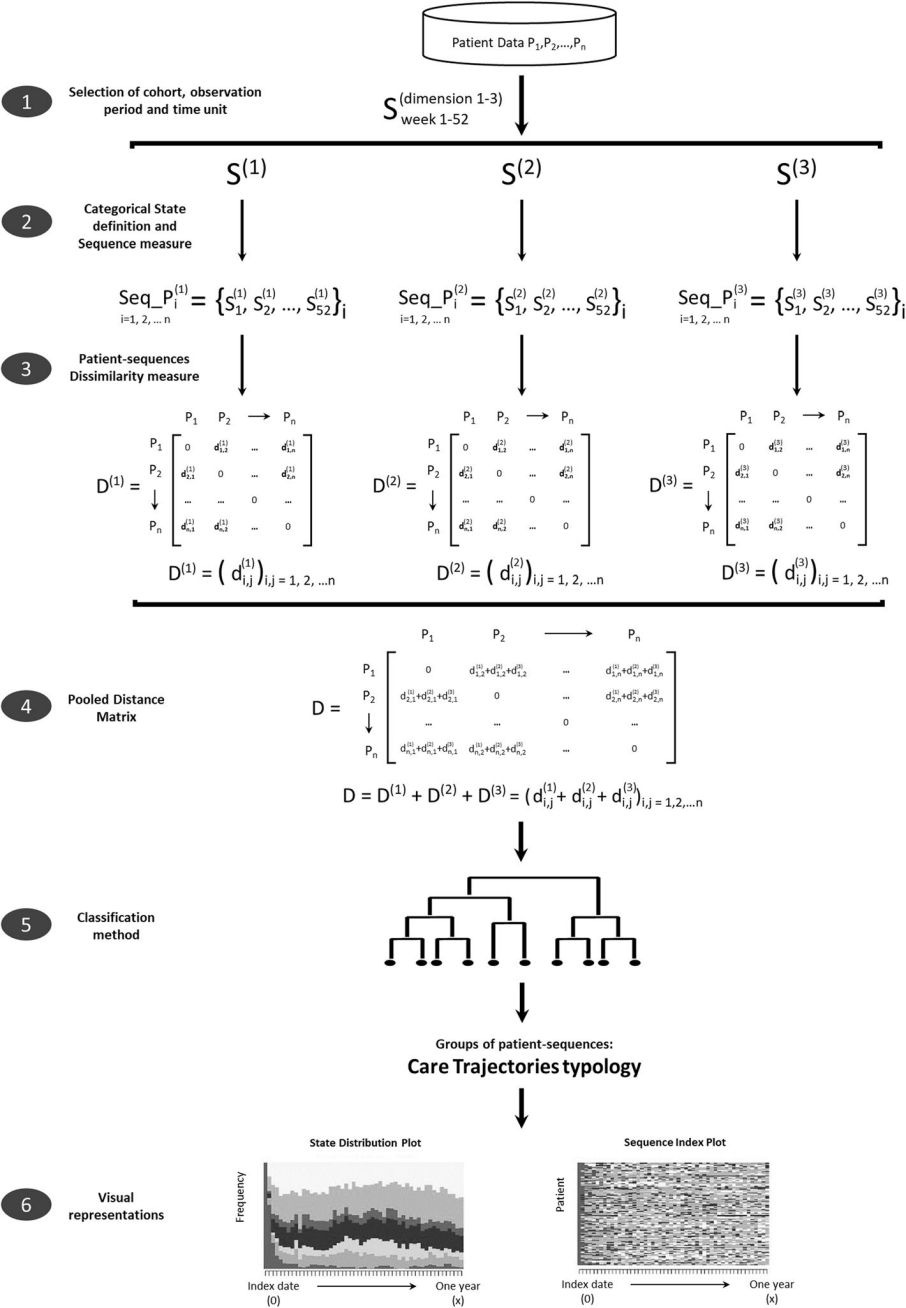
Three-dimensional state sequence analysis diagram: example of week as time unit. The main steps of this multidimensional modified version of SSA were to: Step 1) define the time unit for state sequences analysis (e.g. weeks); Step 2) for each of the three dimensions, select categorical states, specify their priorities and measure the state sequences to generate patient-sequences; Step 3) for each of the three dimensions, calculate the distance between each pair of patient-sequences using an appropriate dissimilarity measure method, resulting in three distance matrices; Step 4) calculate a pooled distance matrix by summing the three dimension-specific matrices; Step 5) based on the pooled distance matrix calculated in step 4, choose and apply a classification method resulting in groups of distinct patient-sequences; and finally 6) display results by visual representations offered by SSA for interpretation

To characterize the typology of CTs (homogeneous groups of CTs), we used a state sequence analysis (SSA) [39]. This method was specifically developed to analyse sequential data [24–26]. Because of the multidimensional nature of CTs (the where, why, and which dimensions), we used a modified version of SSA (Fig. 2). The main steps of this multidimensional modified version of SSA were to: 1) define the cohort of patients, the observation period and the time unit (e.g. days, weeks, months); 2) for each of the three dimensions, select categorical states, specify their priorities as many are not mutually exclusive (e.g. primary care consultation, emergency visit and urgent hospitalization occurring in the same time unit) and measure the state sequences to generate patient-sequences; 3) for each of the three dimensions, calculate the distance between each pair of patient-sequences using an appropriate distance or dissimilarity measure method, resulting in three distance matrices; 4) calculate a pooled distance matrix by summing the three dimension-specific matrices; 5) based on the pooled distance matrix calculated in step 4, choose and apply a classification method resulting in groups of distinct patient-sequences - the CT typology; and finally 6) display results by visual representations offered by SSA to interpret the CT typology [32].

For this study, we measured CTs in the year following the index hospital admission (index date) and chose “weeks” as the time unit (step 1). We defined the following dimension-specific states (step 2): A) the “where” dimension, i.e., the type of care setting or unit patients consulted. Seven possible states, in priority order: hospital (urgent) (state 1), ED (state 2), hospital (non-urgent) (state 3), outpatient clinic (state 3), primary care or private clinic/other (state 4), consultation in CLSC (state 5), and a state for “no healthcare utilization” (state 7); B) the “why” dimension, i.e., the reason for the consultation. Four possible states, in priority order: respiratory disease (state 1), CVD (state 2), other reason (state 3), and a state for “no healthcare utilization” (state 4); C) the “which” dimension, i.e., the type of physician consulted. Height possible states, in priority order: pulmonologist (state 1), cardiologist (state 2), internist (state 3), other MD specialist (state 4), GP (state 5), respiratory therapists in CLSC (state 6), nurse in CLSC (state 7), and a state for “no healthcare utilization” (state 8). Then, for each patient, we defined three CT sequences (one for each dimension) consisting of 52 states (from 1 to 7 for the where dimension, from 1 to 4 for the why dimension, and from 1 to 8 for the which dimension), one for each time unit of follow-up after index date (52 weeks). In case a patient has several states during the same unit of time (e.g. urgent hospitalization and consultation in CLSC during the same week), each state was given a priority (in the same order as listed above, e.g. state 1 hospital (urgent) has the priority over all other states).

In step 3, we chose optimal matching, a method largely used in social sciences [32, 40–42] to measure the distance (or dissimilarity) between patients’ CT sequences for each of the three dimensions. For each pair of CT sequences, this method measures the minimal cost of transforming one sequence into the other. This minimal cost constitutes the “distance” between two CT sequences (or patients). In optimal matching, only three kinds of modifications are allowed: substitution, deletion, or insertion. For the primary analysis, we chose a deletion/insertion cost of 1 and a substitution-cost matrix based on the estimated transition rates, the rational being to set a high cost when changes between two states are seldom observed and lower cost when they are frequent [32]. Three distance matrices, one for each dimension, were created at the end of this step. We explored another substitution-cost matrix in sensitivity analyses.

In step 4, we calculated a pooled distance matrix between CT sequences as the sum of the three dimension-specific distances to propose a unique typology of CTs that accounts for all three dimensions. Hence, two CT sequences that are similar on all three dimensions will have a small sum of dimension-specific distances. Conversely, two CT sequences dissimilar on all three dimensions will have a large sum of dimension-specific distances.

Then, based on this pooled distance matrix, we performed a hierarchical cluster analysis (HCA) in step 5 to classify similar CTs (or patients with similar CTs) [40], i.e., patients with similar sum of dimension-specific distances were classified in the same group. In HCA, each patient starts in his own cluster, and then pairs of clusters are merged as one moves up the hierarchy, until all patients are combined in a unique group. The Ward’s linkage criterion, also largely used in social sciences with dissimilarity measures [25, 40], was chosen to find the pair of clusters that lead to the minimum increase in total within-cluster variance after merging. The choice of the optimal number of groups or clusters was guided on statistical criteria (sum of squares or inertia).

Finally, to interpret and visualize the CT types (step 6), we benefited from the various visual representations offered by SSA. Among them, State Distribution Plots show the distribution of states for each time unit point, and Sequence Index Plots use line segments to show how individuals move from one state to another over time, each line representing an individual’s CT sequence. Once each patient was classified in a specific cluster (with similar CTs), we compared covariables between groups using the usual descriptive statistics (Chi-2 test, t-test, Kruskal Wallis test).

Sensitivity analyses: Since optimal matching offers the opportunity to assign different “costs” to different modifications, we also explored the impact of using different costs matrices. This was done using weeks as the time unit. We also explored other different time units (days and months).

SSA was performed using the TraMineR package in R [43]. All other analyses were performed using SAS 9.4.

## Results

After excluding patients with cancer and those institutionalized, 3197 patients living in Quebec and aged between 40 and 84 years were hospitalized for COPD in 2013 for the first time with a 3 years washout period. Among these 3197 patients, 616 (19.3%) died during the 1-year follow-up period (Table 1), including 147 (4.6%) during index hospitalization. After removal of patients encountering these exclusion criteria, the study cohort included 2581 patients (Fig. 1). Compared to survivors, 1-year mortality was associated to male and older patients, with more comorbidities and higher degree of severity at the index hospitalization, as indicated by higher length of stay, higher levels of intensity of resource used (NIRRU) and high to extreme clinical severity index (Table 1).

**Table 1:** Comparison between deceased patients and survivors

	Total *n* = 3197	Deceased *n* = 616	Survivors (Study cohort) *n* = 2581	*P*-Value
Sex, n (%)				<.0001
Female	1673 (52.3)	278 (45.1)	1395 (54.0)	
Male	1524 (47.7)	338 (54.9)	1186 (46.0)	
Age, mean (SD)	72.1 (8.6)	74.4 (7.9)	71.6 (8.7)	<.0001
Rurality,^a^n (%)				0.7778
Metropolitan area	1855 (58.8)	351 (59.8)	1504 (58.5)	
Small town	540 (17.1)	101 (17.2)	439 (17.1)	
Rural area	761 (24.1)	135 (23.0)	626 (24.4)	
Material Deprivation,^b^ n (%)				0.1771
Quartile 1	449 (15.0)	96 (17.3)	353 (14.4)	
Quartile 2–3	1510 (50.3)	277 (50.0)	1233 (50.3)	
Quartile 4	1045 (34.8)	181 (32.7)	864 (35.3)	
Social Deprivation,^b^ n (%)				0.5804
Quartile 1	529 (17.6)	91 (16.4)	438 (17.9)	
Quartile 2–3	1434 (47.7)	262 (47.3)	1172 (47.8)	
Quartile 4	1041 (34.6)	201 (36.3)	840 (34.3)	
GP affiliation, n (%)	2242 (70.1)	449 (72.9)	1793 (69.5)	0.0956
Combined CI, median (IQR)	4 (2–6)	5 (2–8)	3 (1–6)	<.0001
Length of stay, median (IQR)	5 (3–9)	6 (3–13)	5 (3–8)	<.0001
NIRRU, mean (SD)	1.22 (1.20)	1.58 (1.56)	1.13 (1.08)	<.0001
Severity index, n (%)				<.0001
Weak	470 (14.7)	36 (5.8)	434 (16.8)	
Moderate	1186 (37.1)	173 (28.1)	1013 (39.2)	
High	1132 (35.4)	254 (41.2)	878 (34.0)	
Extreme	409 (12.8)	153 (24.8)	256 (9.9)	

^a^Missing: *n* = 41

^b^Missing: *n* = 193

**Fig. 3.**
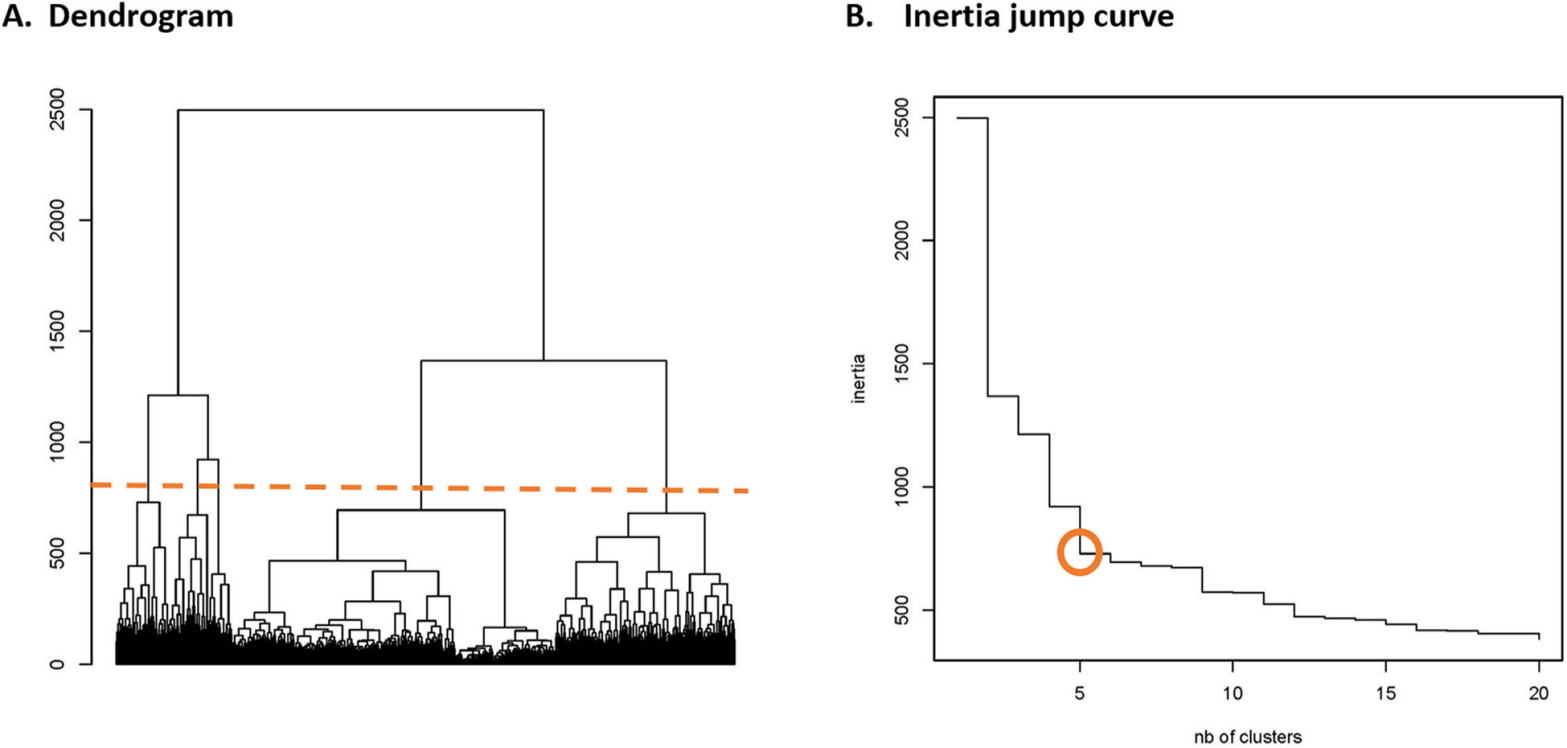
Hierarchical cluster analysis (HCA) - Dendrogram (**a**) and Intertia jump curve (**b**) for state sequences by week. **a** Patients with similar sum of dimension-specific distances were classified in the same group. In HCA, each patient starts in its own cluster, and then pairs of clusters are merged as one moves up the hierarchy, until all patients are combined in a unique group. The Ward’s linkage criterion was chosen to find the pair of clusters that leads to minimum increase in total within-cluster variance after merging. **b** The choice of the optimal number of groups or clusters was guided on statistical criteria (sum of squares or inertia)

**Fig. 4:**
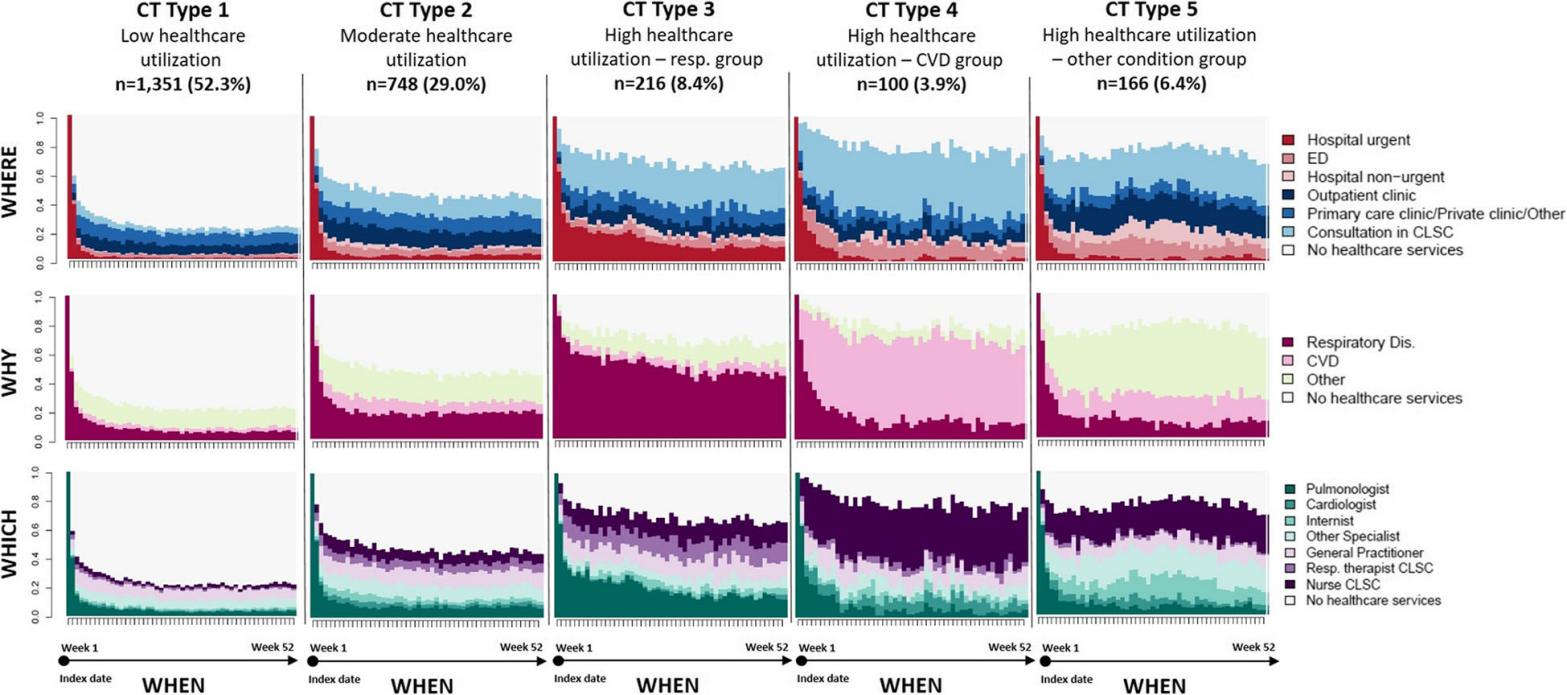
State Distribution Plots of CT typology by dimension (where, why and which). State Distribution Plots show the distribution of states for each time unit point (52 weeks)

**Fig. 5:**
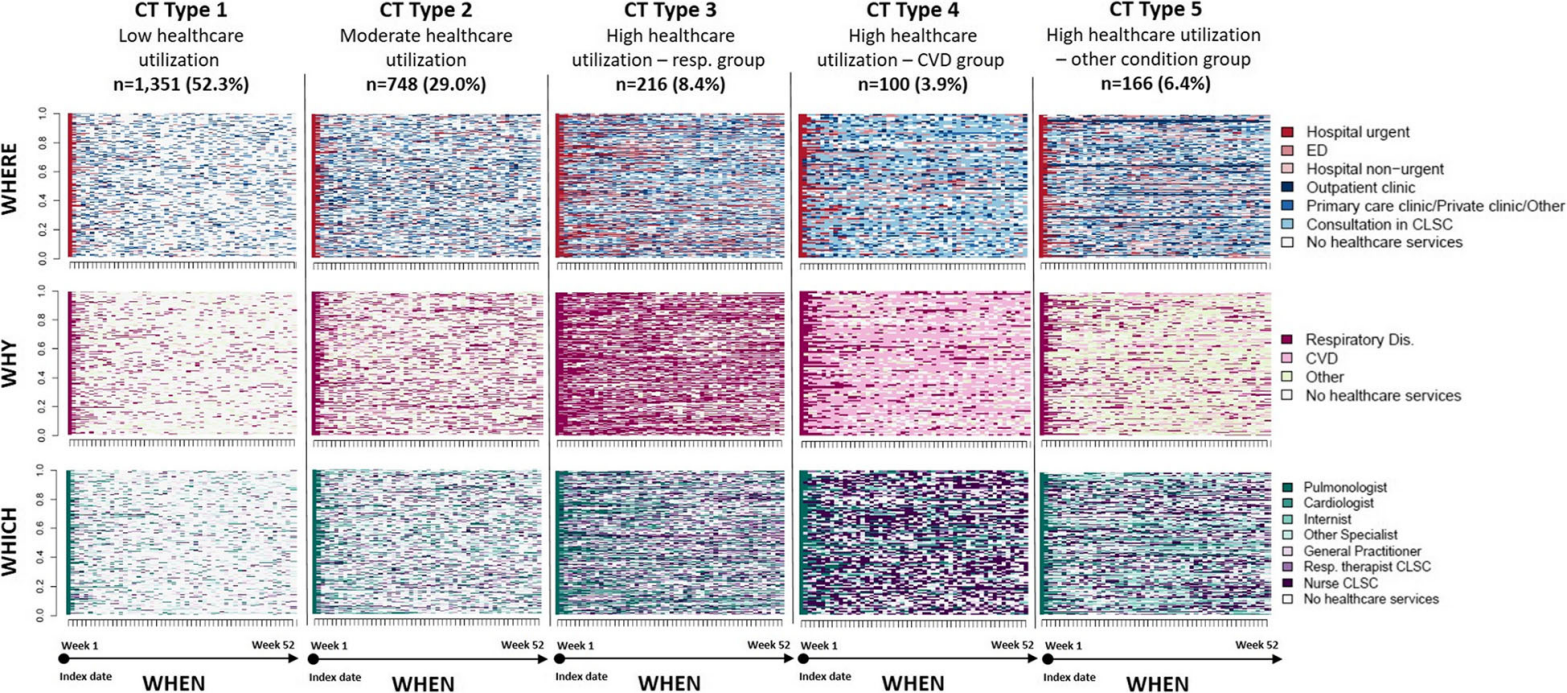
Sequence Index Plots of CT typology by dimension (where, why and which). In Sequence Index Plots, each line represents an individual’s CT sequence

**Fig. 6:**
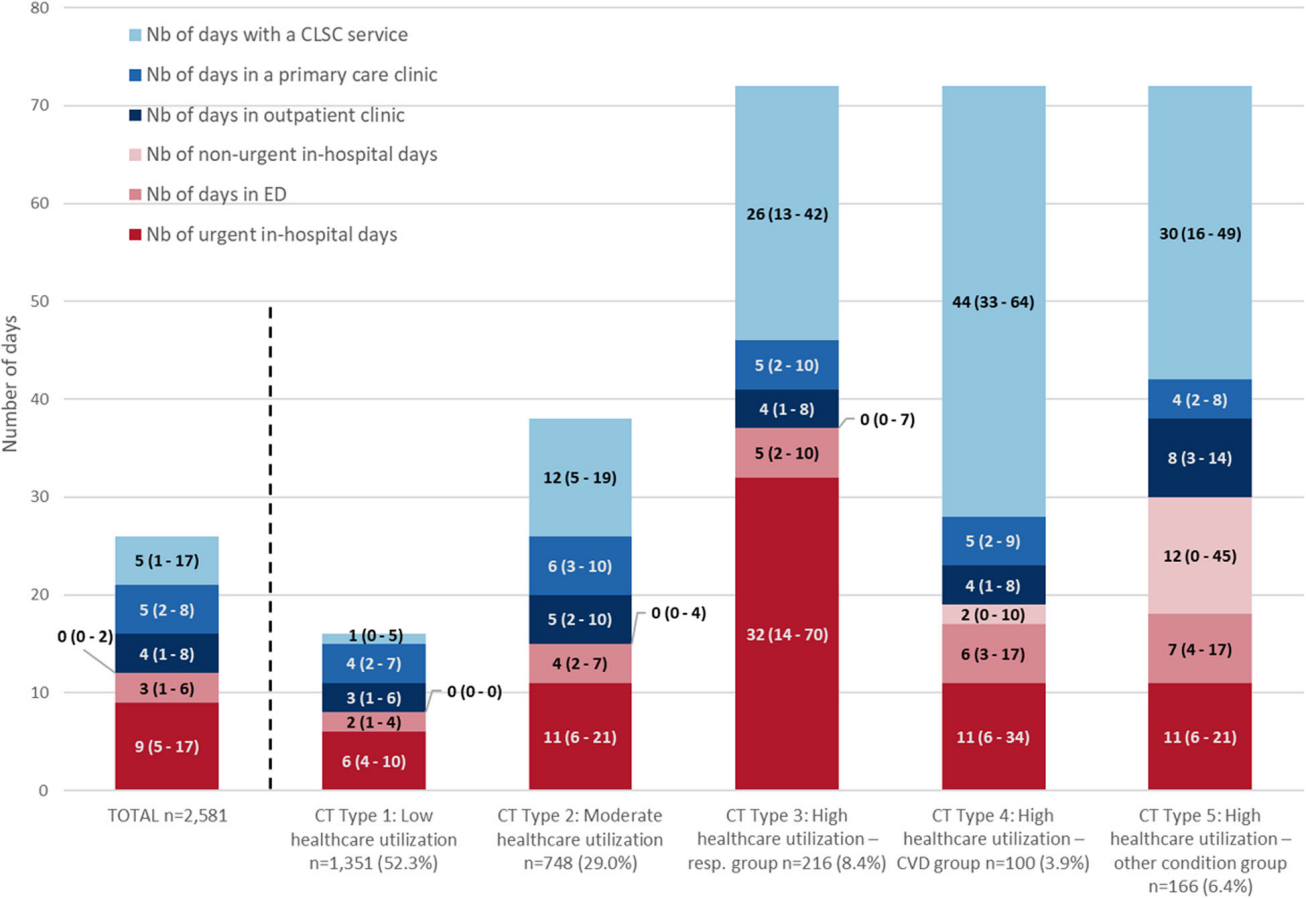
Median (quartiles) number of days spent in each care setting of consultation by CT typology

**Fig. 7:**
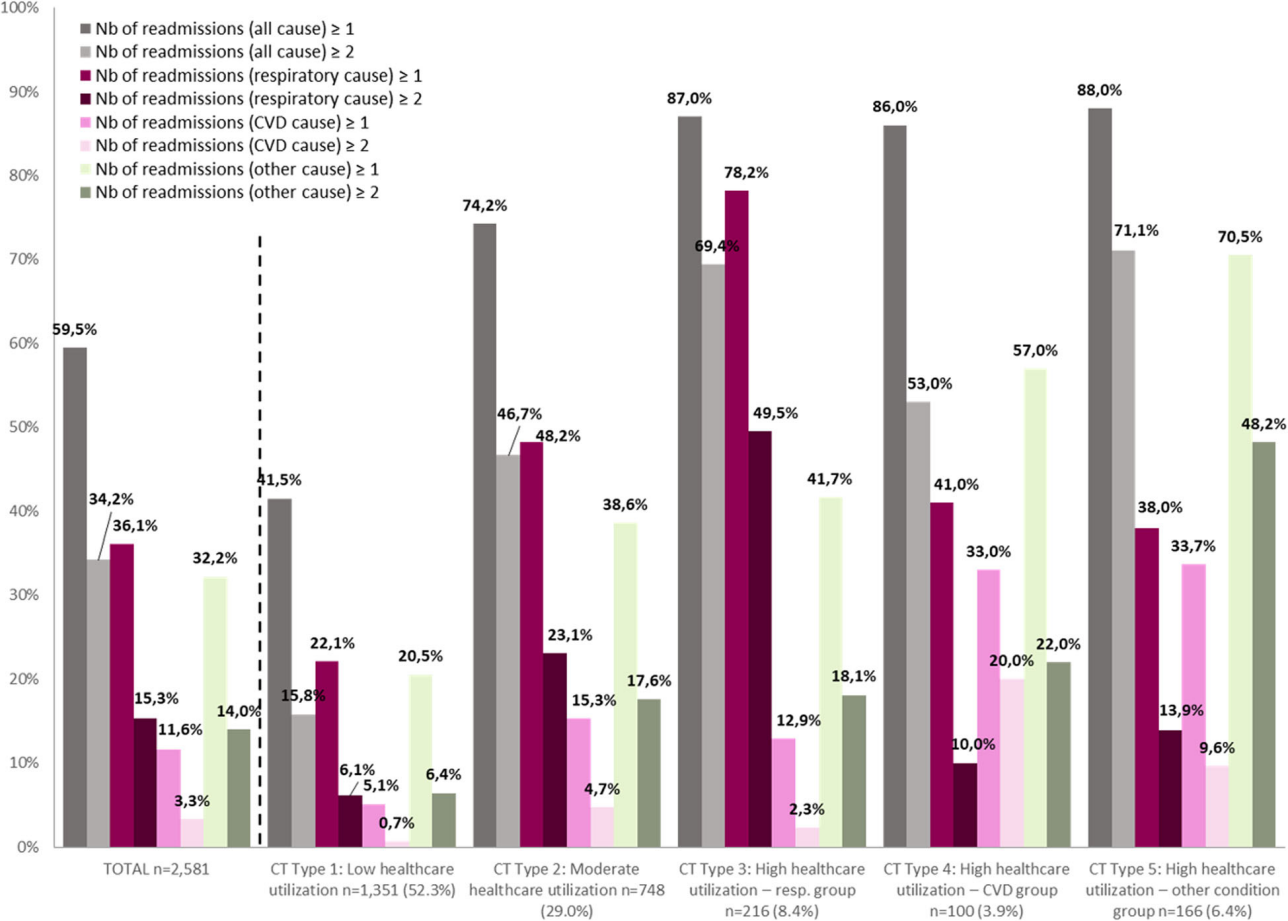
Hospital readmissions in the year following index date by cause and CT typology

In the SSA analyses by week, patients with “similar” CTs were classified into five clusters (Fig. 3a, b) resulting in the CT typology. For each CT type (Type 1 to 5) and dimension (where, why and which), Figs. 4 and 5 present the state distribution plots and the sequence index plots, respectively. The former plots (Fig. 4) present, for each week of follow-up, the proportion of patients in each state, while the latter plots (Fig. 5) present the patients’ state sequence over the year, i.e. each line represents an individual CT sequence. Figure 6 presents the median number of days spent in each care setting by CT type, while Fig. 7 presents the number of hospital admissions during follow-up by CT typology. The combination of Figs. 4, 5, 6 and 7 helps to interpret each CT type.

The CT Type 1 (*n* = 1351, 52.3%) consists of patients with the lowest healthcare utilization; the CT Type 2 (*n* = 748, 29.0%) consists of patients with moderate healthcare utilization, with a slightly higher number of days spent in each care setting than those of the total cohort; the CT Type 3 (*n* = 216, 8.4%) consists of patients with high healthcare utilization for respiratory causes, with more frequent urgent or “unplanned” rehospitalizations for respiratory diseases, more noticeable in the first half of the follow-up year, higher number of urgent in-hospital days, and seen by pulmonologists and respiratory therapists; the CT Type 4 (*n* = 100, 3.9%) consists of patients with high healthcare utilization for CVD causes, with consultations mainly related to CVD, with an important number of services delivered by nurses at CLSCs; the CT Type 5 (*n* = 166, 6.4%) consists of patients with high healthcare utilization mainly for other reasons than respiratory or cardiovascular (the ‘other conditions group’), with frequent ED visits, ‘planned’ or non-urgent hospitalizations, and consultations in outpatient clinics and CLSCs.

Results on hospital readmissions, as important CT attributes (Fig. 7), show that almost 60% of patients had at least one hospital readmission in the year following their index hospitalization: 36.1% were readmitted for a respiratory cause, 11.6% for a CVD cause, and 32.2% for another cause. The readmission rate for respiratory diseases differed greatly between CT types, varying from 22.1% in CT Type 1 to 78.2% in CT Type 3. Also, a non-negligible proportion (15.3%) of patients had at least two respiratory readmissions, with nearly half in the CT Type 3. Almost 75% of patients of the moderate healthcare utilization group (CT Type 2) were readmitted to a hospital, including 48.2% for respiratory diseases.

The CT types differ also in terms of patients’ characteristics (Table 2). Patients in CT Type 4 (High healthcare use - CVD group) were older, came from small towns or rural areas in a higher proportion, had more comorbidities, and had a more severe illness condition at the index hospitalization, shown by higher length of stay, intensity of resources used (NIRRU) and clinical severity index. Patients in CT Type 3 (High healthcare use – respiratory group) and CT Type 5 (High healthcare use – other condition group) had higher index hospital length of stay and intensity of resources index than the other groups, but were similar in terms of patients’ characteristics, although those in CT Type 5 came from rural areas in a lower proportion than the other groups. Patients with the lowest healthcare use, grouped in CT Types 1 and 2, were younger, had fewer comorbidities, and had a less severe index hospitalization (lower length of stay, lower NIRRU and lower severity index).

**Table 2:** Characteristics of the study cohort by CT typology

	CT Type 1 Low healthcare use	CT Type 2 Moderate healthcare use	CT Type 3 High healthcare use – respiratory group	CT Type 4 High healthcare use – CVD group	CT Type 5 High healthcare use – other condition group	*P*-Value
	***n*** **= 1351 (52.3%)**	***n*** **= 748 (29.0%)**	***n*** **= 216 (8.4%)**	***n*** **= 100 (3.9%)**	***n*** **= 166 (6.4%)**	
Sex, n (%)						0.1605
Female	700 (51.8)	416 (55.6)	123 (56.9)	60 (60.0)	96 (57.8)	
Male	651 (48.2)	332 (44.4)	93 (43.1)	40 (40.0)	70 (42.2)	
Age, mean (SD)	70.7 (8.8)	71.9 (8.2)	72.7 (8.3)	76.8 (6.1)	72.3 (9.7)	<.0001
PPDIP status, n (%)						<.0001
Not admissible	178 (13.2)	67 (9.0)	17 (7.9)	x	x	
Admissible – regular	414 (30.6)	265 (35.4)	69 (31.9)	x	x	
Admissible – GIS/LRFA	759 (56.2)	416 (55.6)	130 (60.2)	74 (74.0)	109 (65.7)	
Rurality,^a^ n (%)						0.0262
Metropolitan area	781 (58.0)	444 (59.6)	128 (59.5)	49 (49.0)	102 (63.0)	
Small town	234 (17.4)	118 (15.8)	33 (15.4)	17 (17.0)	37 (22.8)	
Rural area	332 (24.6)	183 (24.6)	54 (25.1)	34 (34.0)	23 (14.2)	
Material Deprivation,^b^ n (%)						0.3034
Quartile 1	187 (14.4)	110 (15.5)	30 (15.0)	8 (8.5)	18 (12.2)	
Quartile 2–3	646 (49.8)	370 (52.1)	94 (47.0)	44 (46.8)	79 (53.4)	
Quartile 4	465 (35.8)	230 (32.4)	76 (38.0)	42 (44.7)	51 (34.5)	
Social Deprivation,^b^ n (%)						0.0083
Quartile 1	249 (19.2)	125 (17.6)	31 (15.5)	18 (19.2)	15 (10.1)	
Quartile 2–3	607 (46.8)	351 (49.4)	100 (50.0)	52 (55.3)	62 (41.9)	
Quartile 4	442 (34.0)	234 (33.0)	69 (34.5)	24 (25.5)	71 (48.0)	
GP affiliation, n (%)	931 (68.9)	515 (68.8)	154 (71.3)	77 (77.0)	116 (69.9)	0.4995
Combined CI, median (Q1 – Q3)	2 (0–5)	3 (2–6)	4 (2–7)	6 (4–9)	5 (3–8)	<.0001
Length of stay, median (Q1 – Q3)	4 (2–7)	5 (3–9)	7 (4–13)	6 (3–11)	7 (4–11)	<.0001
NIRRU, mean (SD)	0.99 (0.76)	1.18 (0.91)	1.56 (2.03)	1.36 (1.23)	1.41 (1.72)	<.0001
Severity index, n (%)						<.0001
Weak-Moderate	833 (61.7)	412 (55.1)	108 (50.0)	31 (31.0)	63 (38.0)	
High-Extreme	518 (38.3)	336 (44.9)	108 (50.0)	69 (69.0)	103 (62.0)	

^x^Suppressed to meet the confidentiality requirements

^a^Missing: *n* = 12

^b^Missing: *n* = 131

### Sensitivity analyses

To see if the results were sensitive to the matrix of substitution cost used in the SSA, we repeated the analysis with a matrix of constant costs. As in the main analysis with transition rates costs, three high healthcare users’ groups emerged, one constituted of patients with respiratory consultations, one with CVD consultations, and one with consultations for other reasons (Supplementary Figure 1). To see if the results were sensitive to the time unit used in the SSA, we reran the analyses by months and days instead of weeks. Results by months were similar to those obtained in the SSA analysis by weeks, with three high healthcare users groups: respiratory, CVD, and other reasons (Supplementary Figure 2). However, results by days produced a different CT typology (Supplementary Figures 3 and 4**)**: two “low” healthcare utilization patterns in day-based CT Types 1 and 2 shared by almost 90% of patients, and two tiny clusters of very high healthcare utilization patterns in day-based CT Types 4 and 5, shared by 1.4 and 1.3% of patients respectively. The day-based CT Type 4 displays mainly urgent hospitalizations for respiratory causes in the first half of the follow-up year, while the CT Type 5 displays mainly non-urgent hospitalizations and use of CLSC services, related mostly to conditions other than respiratory. Unlike week and month-based typology of CTs, care use for CVD diagnosis does not emerge clearly in day-based typology.

## Discussion

### Observed versus expected care trajectories

Patients with a first hospitalization for COPD had been grouped into five structurally distinct types of one-year ‘post-acute’ Care Trajectories. At first glance, the emerging CT typology is somewhat reassuring. First, the intensity of care use seems to be related more to the initial patients’ demographic factors and the severity of their condition, rather than prominent socioeconomic inequalities. Second, results revealed that the most common CT type is the Type 1 “low healthcare utilization” shared by more than 50% of patients, followed by the Type 2 “moderate healthcare utilization” shared by nearly 30% of patients. Third, as expected, the most discriminative factors associated with “high healthcare utilization” patterns, revealed by CT Types 3 to 5, were related to increased age, comorbidities, and the illness condition of patients at the index hospitalization, measured by length of stay, intensity of resources used and clinical severity index. Fourth, no manifest or considerable socioeconomic inequalities in patterns of care use were observed, except for patients of the CT type 4, which came more frequently from rural areas, and were more frequently admissible to the public prescription drug insurance plan with last-resort financial assistance. Finally, the emerging multidimensional typology of CTs shows a connection between the “why”, “which” and “where” dimensions, which seems consistent with the real-world practice. For example, state distribution plots of CTs show that among the “high healthcare utilization” groups, the CT Type 3, the “respiratory group”, had more frequent urgent or “unplanned” rehospitalizations for respiratory diseases, more noticeable in the first half of the follow-up year, consulted mostly pulmonologists, at a hospital or an outpatient clinic, and received health services from respiratory therapists at ambulatory care settings. On the other hand, the CT Type 4, the “CVD group”, had more frequent emergency visits, consulted more cardiologists at ED, hospital or outpatient clinics, and received more health services from nurses at ambulatory care setting. Non-urgent hospitalizations were mostly observed in patients of the CT Type 5, the “other conditions group”, which had more consultations with other categories of specialists at outpatient clinics. Results also show that outpatient and primary care were largely used by patients with high healthcare utilization, corresponding to patients with a more severe initial illness condition, for which post-acute rehabilitation care services delivered at CLSC settings are the most crucial [6, 44].

In this study, almost 60% of patients had at least one readmission (urgent or non-urgent) in the year following the index hospitalization, 36.1% were readmitted to a hospital for a respiratory cause, and 11.6% for a CVD cause. Although one-year post-acute outcomes after a first hospitalization for COPD are rarely reported, these results are generally in agreement with well-known outcomes for COPD patients: readmissions for respiratory conditions are frequent and comorbidities are common, especially CVD [6, 44].

### State sequences: the time granularity effect

SSA delivered relatively consistent results when imposing constant costs of transitions in distance measures instead of measures based on the inherent data transition rates. Nonetheless, results should be taken with caution, since the choice of time granularity could affect state sequences results at different degrees. The typology of CTs is broadly similar when using weeks or months as the time unit, although the size of clusters differs. However, the emerging typology of CTs differs notably when using the day as the time unit (Supplementary Figures 3 and 4), where almost 90% of patients shared the two “lowest” healthcare utilization patterns day-based CT types 1 and 2, compared to 81% in week-based CTs. The day granularity also reveals two tiny atypical or extreme clusters of very high healthcare utilization patterns: a first one with recurrent urgent hospitalizations in the first half of the follow-up year for respiratory diagnoses (day-based CT Type 4), and a second one with non-urgent hospitalizations and nurse services use in ambulatory care, associated mostly to CVD and other conditions (day-based CT Type 5). Another interesting point about the time granularity effect is that primary care encounters emerge more intensely in month and week-based sequences, compared to sequences with day time granularities. Since many categorical states of healthcare utilization are not mutually exclusive, the priorities of each state needed to be established a priori. As a result, hospital and emergency events arose over ambulatory care visits, and specialists’ consultations arose over primary care providers, regardless of the time granularity. However, week-based sequences allow the emergence of lower-priority states, since the probability of primary care and non-specialist encounters increases when using larger time units. Taking this into consideration, rather than a limit, a change in time granularity offers different complementary perspectives of care use patterns. For instance, some “alarming” day-based CT types arose, which may require additional investigation. In particular, the day-based CT type 4 reveals extreme lengths of stay both in urgent hospitalizations and urgent hospital readmissions for respiratory cause, despite an initial severity index broadly similar to most of the other groups (data not shown).

State distribution plots offer a useful visualization of the CT typology, but as expected, such large sets of complex sequences could be problematic to display by sequence index plots due to overplotting, although certain graph simplification and smoothing techniques could be applied [28, 30]. Nonetheless, displaying sequences at the individual level, the sequence index plot of day-based CTs (Supplementary Figure 3), while “noisy”, offers a view of point events, interval events and transitions, such as physician consultations and healthcare services at primary care settings, hospital length of stay, as well as transitions and gaps, which are undetectable in state distribution sequences plots. Also, graphical representations of some CT attributes (total number of days in each care settings and hospital readmission) and characteristics of the study cohort for each CT type provide valuable complementary information (Table 2, Figs. 6 and 7).

### Implications for evidence-based decision-making

Although evidence-based guidelines regarding interventions in primary care such as early pulmonary rehabilitation and counseling could improve patients’ quality of life, exercise tolerance and dyspnea, the effectiveness of such interventions in preventing rehospitalizations for respiratory causes remains unclear [45–48]. However, for each CT type, the relatively large number of consultations at outpatient, primary care and community-based clinics suggests that access to primary care is adequate. The patterns of healthcare utilization described by our approach could contribute to a better evaluation of the impact of new organizational models of healthcare services according to the patient’s condition and concomitant diseases.

### Strengths and limitations

This study has several notable strengths. First, it uses an exhaustive longitudinal dataset of patients hospitalized for COPD in Quebec. This dataset used linked medico-administrative data from multiple sources which provides a comprehensive picture of healthcare services utilization at both inpatient and outpatient settings, including community-based healthcare services provided in ambulatory care (CLSCs). Moreover, the multidimensional approach of care trajectories allowed the possibility to include as much as 19 states, since these states are partitioned into three distinct sequence-dimensions, thus reducing the complexity of each sequence and avoiding the “overplotting” issue [28, 30, 31, 33]. To our knowledge, this is the first study which proposes a comprehensive perspective of care trajectories, allowing a more intuitive and straightforward examination of the most common shared patterns of care use as a whole.

Results need to be taken with caution nonetheless, since the CT typology emerged from the analysis of a specific cohort of patients with a first hospitalization for COPD (infrequent exacerbations). Also, administrative data has inherent limitations: some important variables related to patients’ individual and clinical attributes, such as severity of COPD, body mass index, smoking status, as well as social or caregiver support, which may considerably affect patients’ health condition and care use, are not routinely collected. There are also limitations related to the choice of the clustering method used in the SSA approach. For example, one possible problem in clustering analysis is that different algorithms may lead to different results. However, the graphical inspection of CT sequences (sequence index plots) in clusters (types) can help to evaluate the quality of a partition. Although not presented in this paper, other techniques exist to help the visualization of complex sequence data, for example by analyzing dissimilarities using multi-dimensional scaling (MDS) and smoothing techniques [28, 49].

## Conclusion

In the field of health service research, SSA is a flexible and promising method to describe and visualize care trajectories of patients with COPD, and this method could be applied to explore CTs of other chronic diseases. Considering all-cause post-acute healthcare utilization, instead of a set of predefined outcomes for a single condition, this approach avoids missing significant parts of healthcare utilization for other health conditions. Using days as the time unit, the proposed SSA approach also offers the opportunity to expose atypical patterns.

Finally, this paper has demonstrated the usefulness of the "6W" multidimensional approach for SSA of care trajectories. Future studies are possible, such as linking key measures of treatments to each CT type as explanatory variables. Along with patients’ characteristics, these additional – not to say crucial – variables would allow a complete exploration of care trajectories, taking into account the “who”, “where”, “why”, “which”, “when”, and “what”.

## Supplementary information

**Additional file 1.**

**Additional file 2.**

**Additional file 3.**

**Additional file 4.**

## Data Availability

The datasets generated and/or analysed during the current study are not publicly available due to individual privacy but are available from the corresponding author on reasonable request.

## Abbreviations

CLSC: Local community service centre
COPD: Chronic obstructive pulmonary disease
CT: Care trajectory
CVD: Cardiovascular disease
ED: Emergency department
GIS: Guaranteed income supplement
GP: General practitioner
HCA: Hierarchical cluster analysis
ICD: International classification of diseases
IQR: Interquartile range
LRFA: Last-resort financial assistance
NIRRU: Niveau d’Intensité Relative des Ressources Utilisées (relative intensity level of resources used)
PPDIP: Public prescription drug insurance plan
RAMQ: Régie de l’assurance maladie du Québec
SSA: State sequence analysis
