# Supplementary figures and images for: Healthcare utilization after a first hospitalization for COPD: a new approach of State Sequence Analysis based on the '6W' multidimensional model of care trajectories
Source: BMC Health Serv Res. 2020 Mar 6;20:177. doi: 10.1186/s12913-020-5030-0 (PMC7059729; doi:10.1186/s12913-020-5030-0)

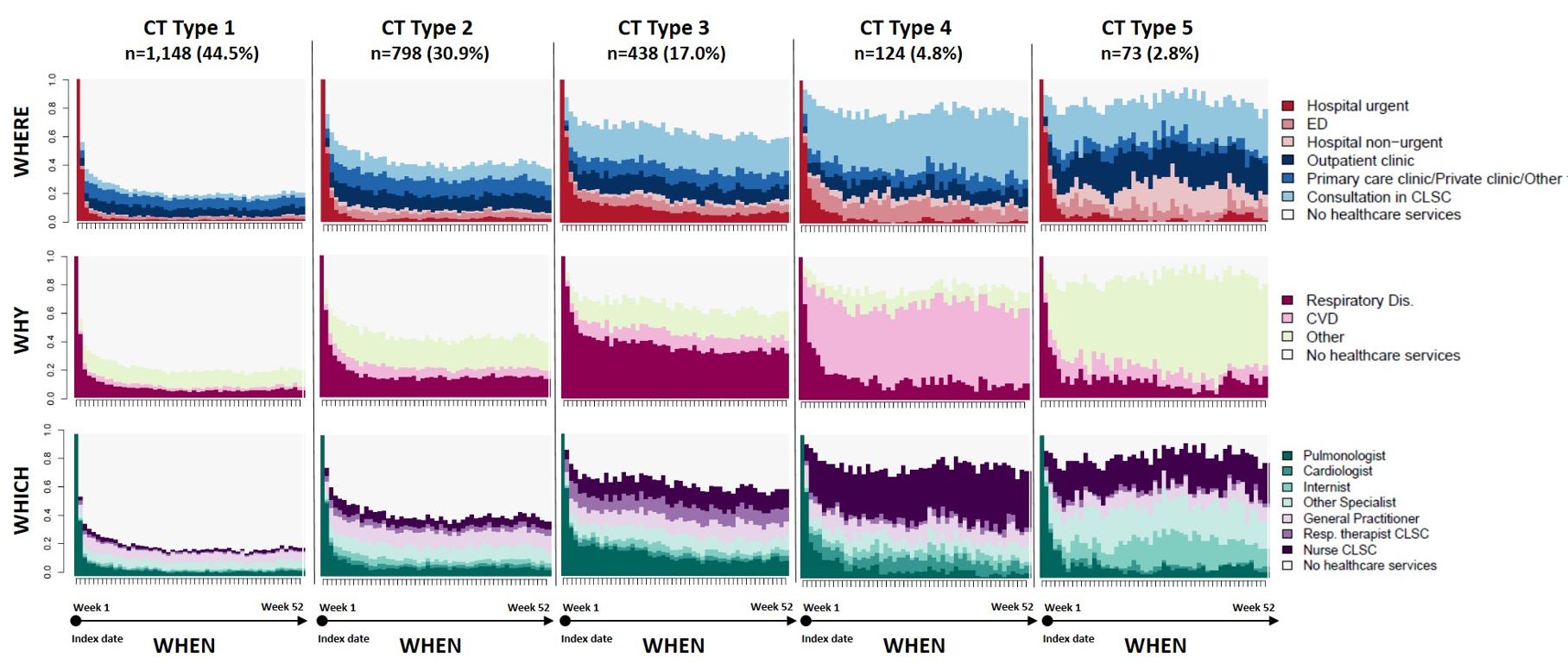

Supplement: Supplementary file 1 — Additional file 1. [file 12913_2020_5030_MOESM1_ESM.png]

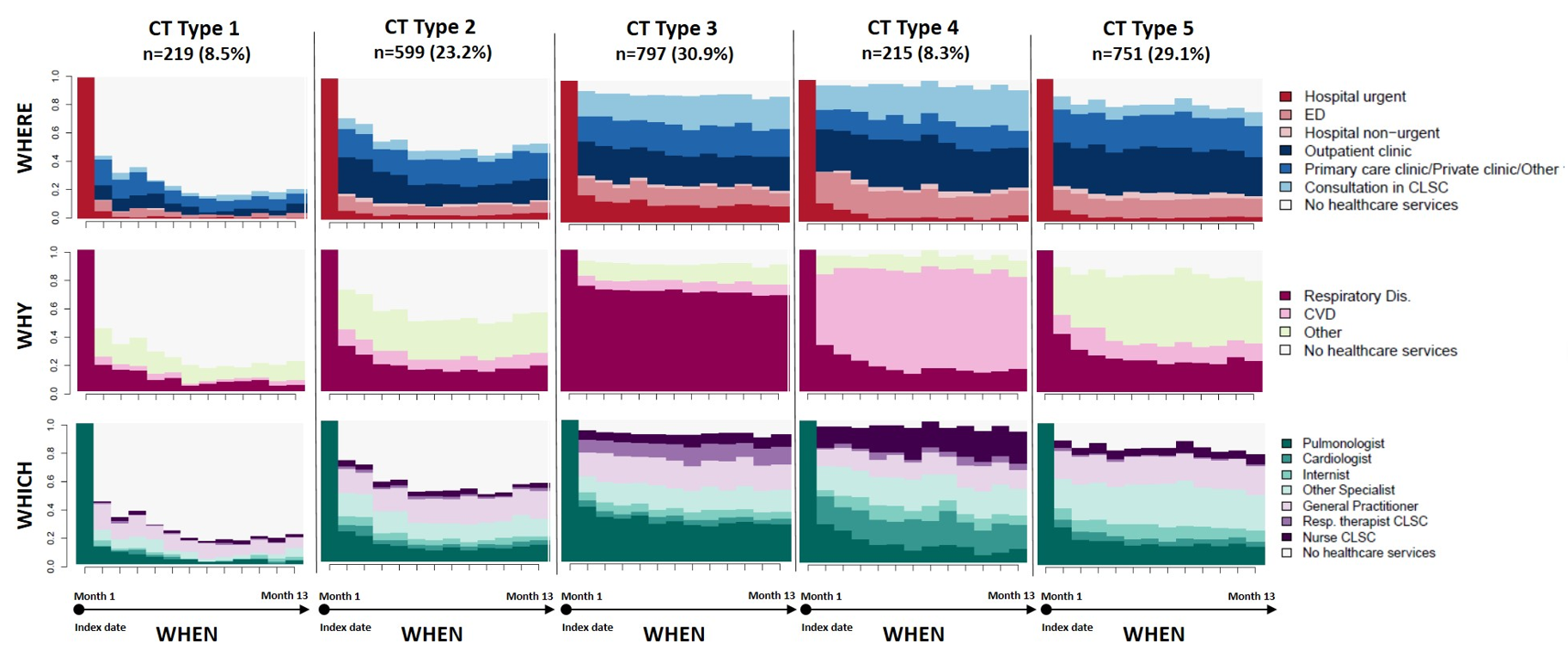

Supplement: Supplementary file 2 — Additional file 2. [file 12913_2020_5030_MOESM2_ESM.png]

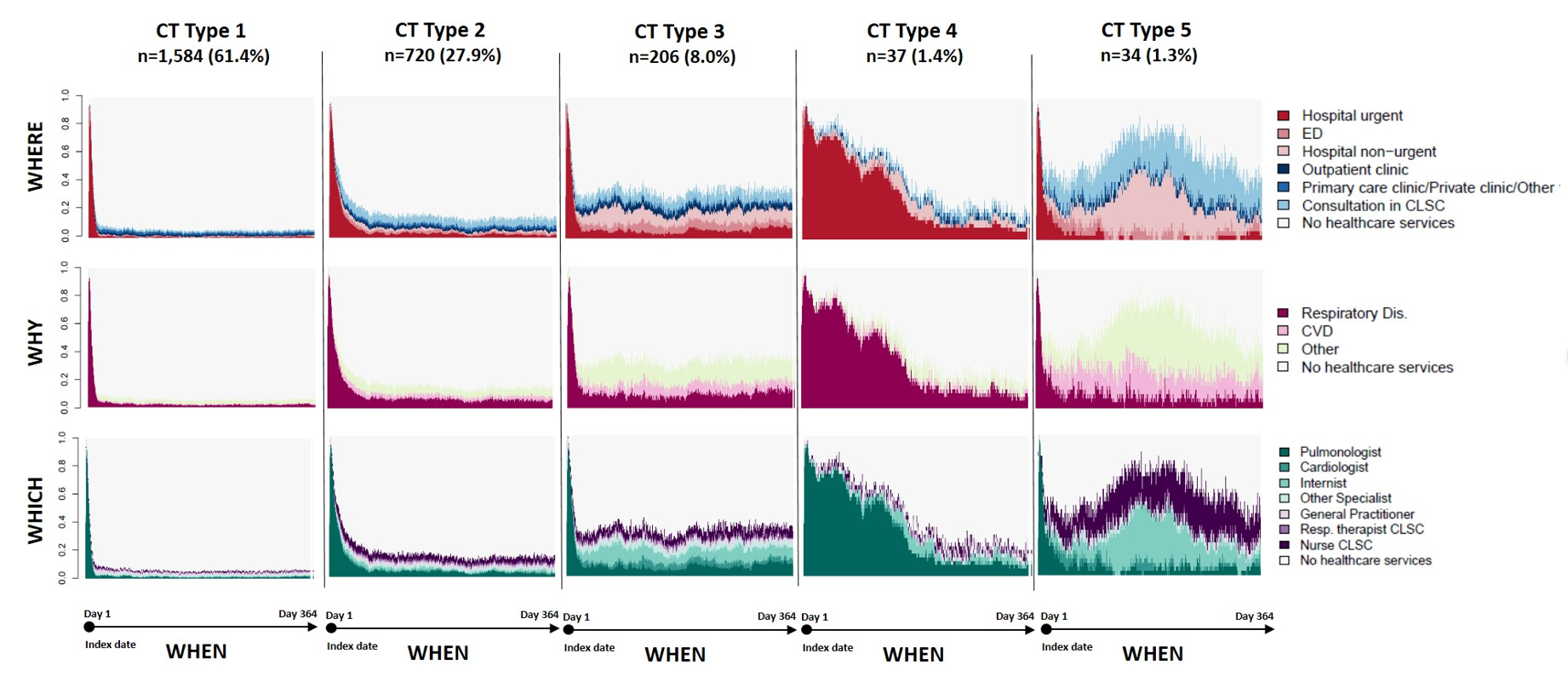

Supplement: Supplementary file 3 — Additional file 3. [file 12913_2020_5030_MOESM3_ESM.png]

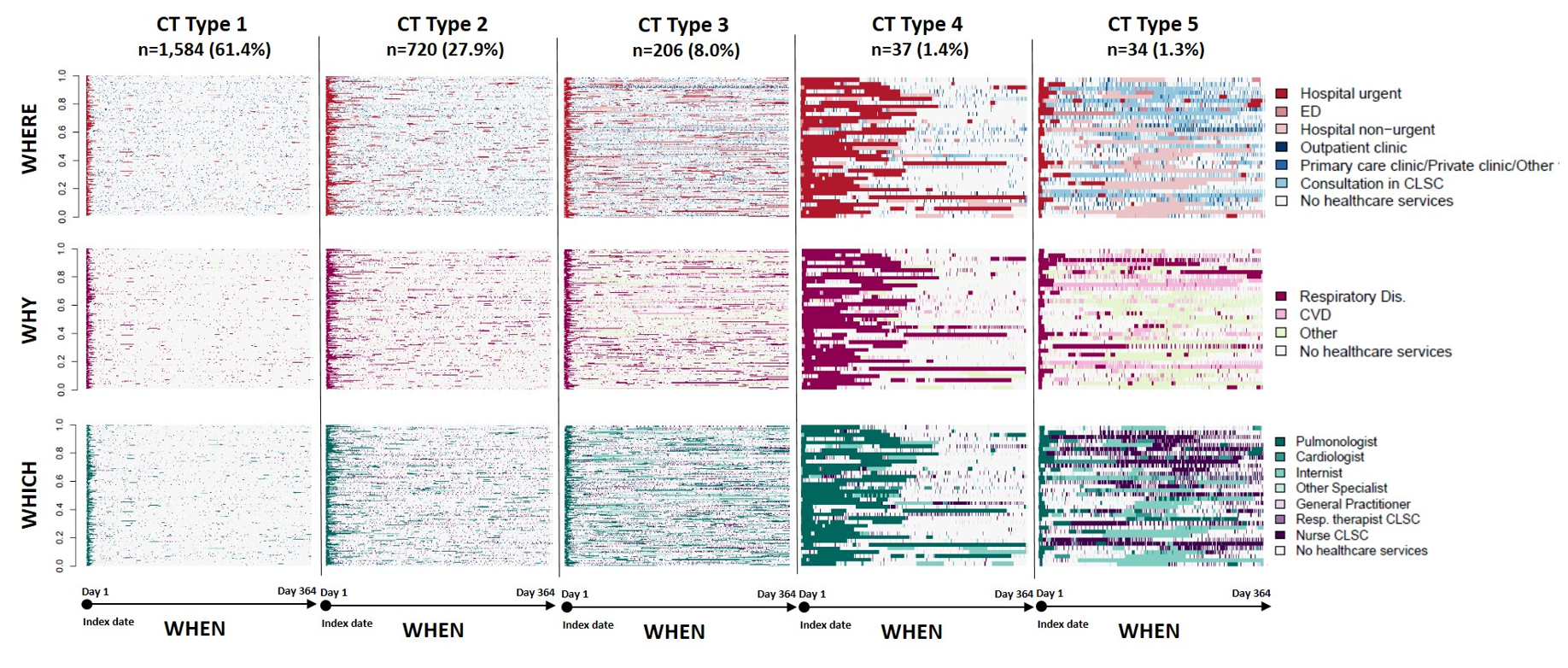

Supplement: Supplementary file 4 — Additional file 4. [file 12913_2020_5030_MOESM4_ESM.png]
